# Abnormal centriolar biomarker ratios correlate with unexplained bull artificial insemination subfertility: a pilot study

**DOI:** 10.1038/s41598-023-45162-8

**Published:** 2023-10-26

**Authors:** Katerina A. Turner, Luke Achinger, Dong Kong, Derek F. Kluczynski, Emily Lillian Fishman, Audrey Phillips, Barbara Saltzman, Jadranka Loncarek, Bo R. Harstine, Tomer Avidor-Reiss

**Affiliations:** 1 Department of Biological Sciences, College of Natural Sciences and Mathematics, University of Toledo, 3050 W. Towerview Blvd, Toledo, OH 43606 USA; 2 Department of Urology, College of Medicine and Life Sciences, University of Toledo, Toledo, OH USA; 3 Laboratory of Protein Dynamics and Signaling, Center for Cancer Research, National Institutes of Health, National Cancer Institute, Frederick, MD USA; 4 Department of Population Health, College of Health and Human Services, University of Toledo, Toledo, OH USA; 5 Select Sires, Inc., Plain City, OH 43064 USA

**Keywords:** Diagnostic markers, Spermatogenesis

## Abstract

The mechanisms underlying male infertility are poorly understood. Most mammalian spermatozoa have two centrioles: the typical barrel-shaped proximal centriole (PC) and the atypical fan-like distal centriole (DC) connected to the axoneme (Ax). These structures are essential for fertility. However, the relationship between centriole quality and subfertility (reduced fertility) is not well established. Here, we tested the hypothesis that assessing sperm centriole quality can identify cattle subfertility. By comparing sperm from 25 fertile and 6 subfertile bulls, all with normal semen analyses, we found that unexplained subfertility and lower sire conception rates (pregnancy rate from artificial insemination in cattle) correlate with abnormal centriolar biomarker distribution. Fluorescence-based Ratiometric Analysis of Sperm Centrioles (FRAC) found only four fertile bulls (4/25, 16%) had positive FRAC tests (having one or more mean FRAC ratios outside of the distribution range in a group’s high-quality sperm population), whereas all of the subfertile bulls (6/6, 100%) had positive FRAC tests (*P* = 0.00008). The most sensitive biomarker was acetylated tubulin, which had a novel labeling pattern between the DC and Ax. These data suggest that FRAC and acetylated tubulin labeling can identify bull subfertility that remains undetected by current methods and may provide insight into a novel mechanism of subfertility.

## Introduction

Unexplained infertility is a major problem in managing and treating reproductive health^1–3^. One out of three couples experiencing infertility have unexplained infertility^4,5^. It is expected that a significant portion of unexplained infertility is due to infertile men who, due to the limitation of current semen analysis methods, appear to have normal semen. Similarly, up to 5% of marketed, genetically elite Holstein bulls used in the artificial insemination (AI) industry have unexplained subfertility that is found only after being released to the market^6,7^. Because of this similarity and the similarity of cattle sperm centrioles' structure, composition, function, and inheritance pattern to human sperm centrioles, cattle subfertility is an important model for human infertility^8^.

Subfertility, a significantly reduced reproduction rate relative to average reproduction rate by most other bulls, is a major obstacle to the profitability of the U.S. meat and dairy industry^9^. Currently, the markers used to diagnose bull subfertility explain ~ 3% of the total variance in artificial insemination field fertility deviations^6^. Bovine sperm is extensively analyzed prior to use and undiagnosed subfertility after analysis points to an unknown male factor and a gap in our understanding of sperm biology. One reason for this deficiency is the absence of a comprehensive list of independent biomarkers that identify defects in distinct properties of sperm, which are essential for selecting bulls with high fecundity^10^. Therefore, there is a need to identify novel biomarkers for male subfertility.

One approach to identify new biomarkers is to study sperm structures that are implicated in sperm function and fertility but are not routinely used for diagnostics, such as the sperm centrioles^11–15^. Identifying biomarkers in bull sperm has the benefit of not only improving the selection of bulls, but it is also a good model for human sperm because they share a similar morphology, both having paddle or pear-like sperm heads with a canonical proximal centriole and an atypical distal centriole in the neck, whereas mice have sickle shaped sperm heads with no centrioles and numerous other embryonic developmental deviations reviewed in^16^. Robust fertility data provided by the bull artificial insemination industry, which examines over 300 (and up to over 100,000) insemination attempts per bull in the field, provide unprecedented reproductive statistics for determining normal biomarker ranges. Therefore, bulls are a valuable model to understanding human sperm biology, while also providing insight for the cattle industry. Yet, bull and human fertility are managed differently as bulls, unlike humans, are selected for fertility, creating challenges in using results from domestic animal testing for human reproductive assistance programs.

Bull fecundity depends on the ability of sperm to travel through the female reproductive tract and the contributions of the sperm components in the early embryo^17^. A sperm component that functions in both processes are the centrioles. They form the sperm tail during spermatogenesis, are dynamic during sperm swimming, organize the microtubule in the zygote, and are essential for accurate cell division^18,19^. These multitude of functions imply that centriole quality could be an indicator of sperm fertility. Interestingly, despite most dividing cells requiring precisely two centrioles, there is only one canonical centriole, the proximal centriole (PC), in the sperm of humans, bovine, and other non-murine mammals; the second centriole is the remodeled atypical spermatozoa distal centriole (referred to as the DC in this paper)^20^. The role of the remodeling, or the centrioles in general, in sperm biology and male fertility is not well understood^8^. Since the centrioles are not examined during routine semen analysis, evaluating the centriole may be a useful independent biomarker for advancing semen analysis^5^. Recently, a high throughput method was developed to assess sperm centrioles, Fluorescence-Based Ratiometric Analysis of Sperm Centrioles (FRAC).

**Figure 1:**
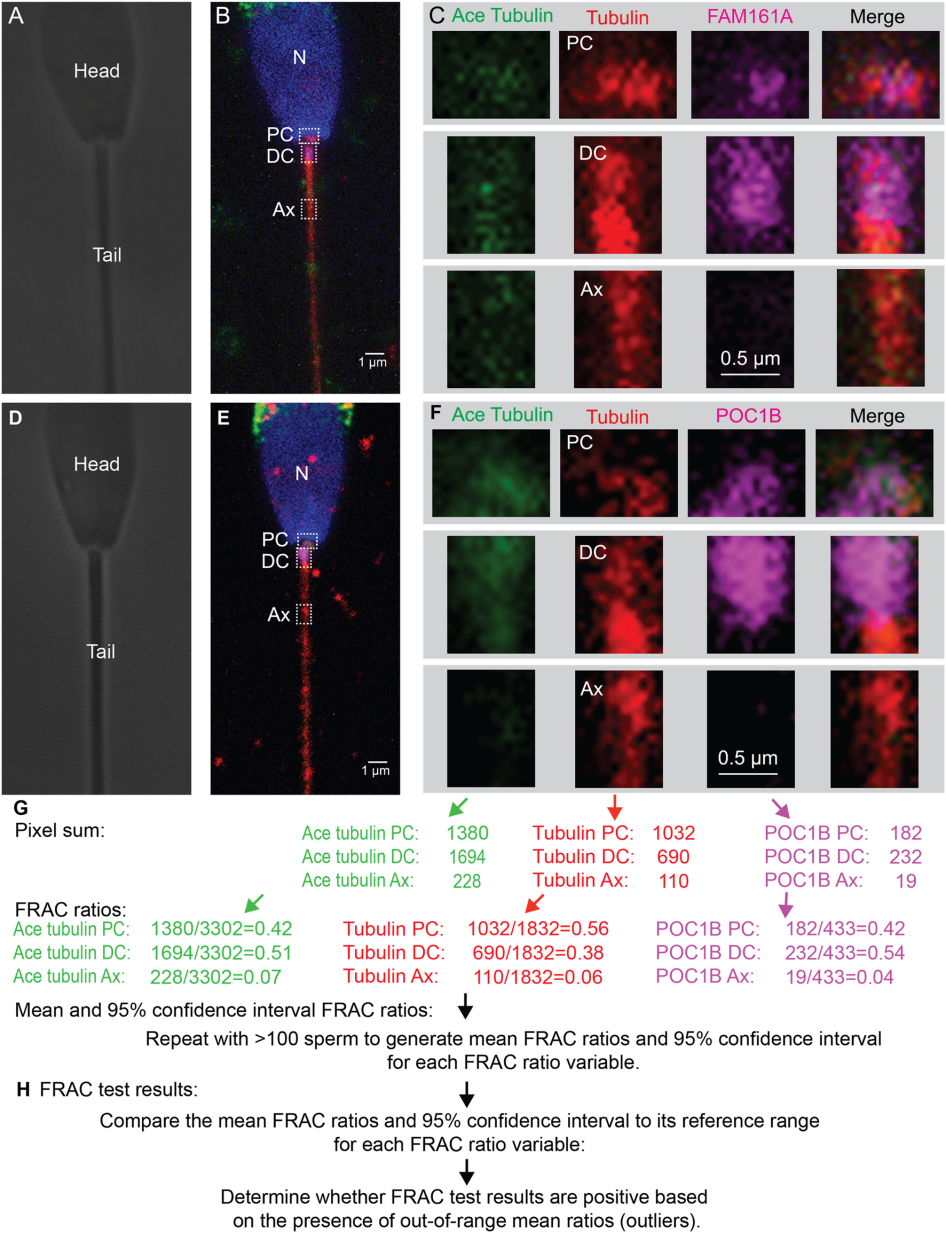
FRAC analysis of bull sperm: (**A**–**B**, **D**–**E**) Transmission (**A** & **D**) and fluorescent (**B** & **E**) images of bovine sperm oriented with the PC on the right side. Three boxes mark the approximate location of the PC, DC, and Ax, insets presented in panel (**C**, **F**). Nucleus (N). **C** & **F**) Higher magnification of the proximal centriole (PC), distal centriole (DC), and axoneme (Ax) of sperm from panel labeled with anti-acetylated tubulin, anti-tubulin, and anti-FAM161A (**C**), or anti-POC1B (**F**) antibodies. Tubulin labeling in the PC and DC is more intense than the nearby axoneme under non-saturating labeling and imaging conditions^20^. (**G**) FRAC ratio calculation starts by quantifying raw intensity values (photon counting) for each of the three different biomarkers from the PC, DC, and Ax ROIs and then, calculating FRAC ratios for each sperm. This is repeated to multiple sperm. These FRAC ratios are averaged together to form a mean FRAC ratio and a 95% confidence interval for an individual bull. (**H**) A bull’s FRAC test starts by comparing the 95% confidence intervals to the reference range for each parameter and counting the number of outliers and their distance from the reference range in SDs. If a bull has a single outlier mean ratio in one of the 12 parameters studied, then its FRAC test is considered to be positive, indicating that the bull has abnormal centrioles.

FRAC determines the intensity ratio of centriolar biomarkers at locations in the sperm neck and mid piece for each sperm (the PC, DC, and Ax). Then a mean ratio 95% confidence interval is calculated for each bull by analyzing over 100 sperm. The 95% confidence interval is compared to a reference population and, if it is outside, then the bull has a positive FRAC test indicating a possible centriole anomaly (Fig. 1). This technique found that patients diagnosed with teratozoospermia (poor morphology) had lower centriole quality^21^, as was suggested earlier by electron microscopy studies^22–32^. Also, FRAC identifies male factor infertility in couples with unexplained infertility^33^. FRAC is a sensitive method for identifying male infertility because it detects changes in relative protein distribution using fluorescent microscopy. Thus, FRAC could stratify centriole quality better than a low throughput assays like electron microscopy, but this requires futher evaluation.

After fertilization, the two centrioles organize the embryonic cytoskeleton and are the progenitors of centriole duplication within the embryo^34^. These centrosomes, via their astral microtubules, bring together the male and female pronuclei and polarize the chromosomes inside the pronuclei^17,35–39^. These centrioles are the only embryonic structures inherited exclusively from the father^40^ and, in many basal animals, they are essential for embryonic development^41–43^. Therefore, we hypothesize that defects in bull centrioles would lead to unsuccessful pregnancies^44^. Indeed, despite high rate of fertilization, cows have a high rate of pregnancy loss reviewed in^45,46^. Fertilization occurs in 80–90% of the cows that are inseminated with high-fertility sperm based on standard semen analysis. However, a significant loss occurs postfertilization, and before maternal pregnancy recognition (~ day 16 postfertilization) and may be due to undiagnosed sperm defects. Similarly, lactating dairy cows, during the first week of gestation, lose 20–50% of pregnancies and during conceptus elongation (Days 8–27) an additional ~ 30% loss occurs. Some of these failures are lactation induced or due to heat stress reviewed in^46,47^. However, some of these failures may also be due to defects in the sperm centrioles that either lead to failure to properly polarize the pronuclei, or inaccurate cell division.

Centriolar biomarkers can be structural proteins or posttranslational modifications^33^. Tubulin is a building block of PC, DC, and Axoneme microtubules reviewed in^48^, and its labeling is more intense in the centrioles than in the axoneme of human sperm^20,21^. Acetylated tubulin is a posttranslational modification of tubulin that makes the microtubules more flexible and stable^49,50^. Its labeling has a similar intensity in the centrioles and the axoneme of bovine sperm^51^. POC1B and FAM161A are luminal centriole proteins^52^ that form the rods of the sperm centrioles but appear to function independently^19^. Their labeling is specific to centrioles and POC1B is essential for male fertility in humans and mice^53^.

A measure of field fertility by artificial insemination in bulls (also referred to as sires) in the USA is Sire Conception Rate (SCR)^54–56^. It is an outcome predictor based on more than 300 inseminations, sometimes from tens of thousands of inseminations. SCR is reported in deviation percentage units from average pregnancy rate within breed generated from more than 500 bulls. Average Holstein successful conception is approximately 34–35%, which is an SCR of 0^57,58^. Using a bull with a significantly lower SCR is time-consuming and costly due to the need for increased rebreeding attempts, extended days open, widened calving intervals, and increased risk of culling when a threshold number of insemination attempts have been reached. Both farmers and the artificial insemination industry are interested in screening out such bulls.

Hidalgo and colleagues (2021) compared 12 bulls with pregnancy rates of 33–52% to 12 bulls with rates of 52–70% after fixed time artificial insemination in Brazil^59^. They found that bulls with greater fertilization capacity have higher percentages of fast and nonlinear spermatozoa (*P* < 0.05). By examining 10 Holstein bulls with high or low SCR, Ortega and colleagues (2018) found that some low SCR bulls produced fewer day 8 blastocysts in IVF using slaughterhouse-sourced oocytes^60^. They also showed some low SCR bulls produced a lower percentage of fertilized oocytes or more degenerated embryos compared to high SCR bulls in super-ovulated heifers.

Because the centriole functions during sperm swimming and embryo development, we hypothesized that centriolar anomalies act as biomarkers for bull subfertility. Therefore, we studied the correlation between sperm centriole protein distribution and SCR by studying fertile and subfertile bulls. We found that some subfertile bulls have abnormal centriole protein distribution that can be identified by FRAC. This finding is the first experimental evidence that bovine sperm centriole anomalies are associated with unexplained subfertility. Furthermore, the utilization of FRAC provides a path to improving artificial insemination efficiency in the short term by weeding out bulls with lower sperm quality and could be used to improve artificial insemination fertility in the long term by selecting bulls with higher sperm quality.

## Methods

The methods utilized in this manuscript are similar to those published before in Jaiswal and colleagues (2022). A few changes were made, mostly to adapt the method from human semen to bovine semen^33^. No live animals were experimented on to generate these data. All experimental protocols were approved by UToledo Biosafety Committee (IBC). All methods were carried out in accordance with UToledo Biosafety Committee (IBC) guidelines. The ARRIVE guidelines (Animal Research: Reporting of In Vivo Experiments) are not applicable as Animals were not used directly in the study.

### Eligibility criteria for semen sample usage

Semen samples from 31 bulls were obtained from Select Sires, Inc. in September of 2019 from available bulls with known, varying SCRs (25 fertile with an SCR > − 3, and 6 subfertile with an SCR < − 3). Semen was collected by Select Sires as described in DeJarnette and colleagues and distributed to us cryopreserved in straws in liquid nitrogen tanks as regularly done in the dairy bull AI industry^6^. Samples in straws were stored in liquid nitrogen until studied by FRAC. FRAC analysis was performed during 2020–2022 and ended once all 31 samples were analyzed. It should be noted that only semen samples collected by Select Sires were used to generate data presented herein.

#### Differential gradient centrifugation, fixation, and attachment to coverslips

To examine the sperm that are likely to participate in fertilization, semen samples were separated into less dense lower-quality and denser higher-quality sperm using differential gradient centrifugation following manufacturer’s instructions (Nidacon, PureSperm). Frozen samples and all necessary PureSperm media were brought to 37 °C in a water bath. Then, 1.0 mL of PureSperm 80% (Nidacon, PS80-100) was placed into a 15 mL conical tube labeled with the bull identifier and pellet. Next, 1.0 mL of PureSperm 40% (Nidacon, PS40-100) was carefully pipetted to create a layer on top of the lower phase. The contents of the sperm straw were emptied into a 1.5 mL tube by cutting both ends using scissors. The ~ 0.5 mL thawed semen sample was then layered on top of the upper phase using a pipette. The conical tube was centrifuged at room temperature for 20 min at 400 × g. Following centrifugation, the middle layer (interface, low-quality sperm) was removed and placed into a second 15 mL conical tube, labeled with the bull identifier and interface. Remaining liquid was removed from the pellet tube without disturbing the pellet (high-quality sperm) and was discarded appropriately, leaving behind the high-quality pellet sperm. Both samples were washed with 2.0 mL PureSperm® Wash (Nidacon, PSW-100) media and were gently resuspended by pipette. Tubes were then centrifuged for 8 min at 250 × g. Enough supernatant was removed from both tubes without disturbing the pellets and discarded appropriately. Both pellets were resuspended with 100 μL of sterile water.

The sperm was chemically fixed by adding 1 mL of – 20 °C methanol to the sperm solution (high-quality or low-quality sperm samples) and the samples were immediately centrifuged for 2 min at 1000 × g. The methanol was pipetted off and 500 μL of sterile water was used to resuspend the sperm. 20 μL of high-quality or low-quality samples were pipetted onto gelatin coated 8 mm round coverslips and allowed to dry completely. Afterwards, the coverslips were stored at − 80 °C until needed. When needed for labeling, they were thawed at room temperature.

Gelatin coated 8 mm round coverslips were generated by immersing in 0.1% v/v gelatin (Sigma Adrich, G7765-250ML) for 10 min at room temperature. Next, the coverslips were placed onto a paper towel and allowed to dry at room temperature. Gelatin coated coverslips were either immediately used or stored at 4 °C.

### Sperm labeling

Coverslips containing sperm from each sample fraction/population were stained at least three independent times. Samples retrieved from − 80 °C storage were first permeabilized with 0.3% Triton X-100 (Sigma-Aldrich, 9002–93-1) in PBS (PBST) for 1 h and blocked with 1% BSA (CHEM-IMPEX INT’L, 00535) in PBST for 30 min (PBSTB). Primary antibodies were diluted in PBSTB according to Table 1 and applied to the coverslip which sat on Parafilm in a humidity chamber, and incubated overnight at 4 °C. The next day, coverslips were first washed in PBST three times for 5 min each. Subsequently, coverslips were incubated at room temperature for 4 h with secondary antibodies and Hoechst diluted in PBSTB according to Table 1. Secondary antibodies were diluted in PBSTB. Next, the coverslips were washed in PBST 3 times for 5 min each. Samples were then washed in PBS 3 times for 5 min each. One drop of Fluoroshield with DAPI (Sigma-Aldrich, F6057) was added to the middle of each slide. Coverslips were then placed over and sealed with clear nail polish. Slides were kept at 4 °C until use.

**Table 1:** Antibodies used in this study for FRAC.

Target	Name, company name, catalog number, batch number	Dilution	Role	RRID
Tubulin	Sheep anti-tubulin, Cytoskeleton, Inc., ATN02	1:600	Primary antibody	AB_10708807
Acetylated tubulin	Mouse anti-Acetylated tubulin, Sigma Aldrich, T7451-200UL	1:70	Primary antibody	AB_609894
POC1B	Rabbit anti-POC1B, Thermo Fisher Scientific, PA5-24,495	1:100	Primary antibody	AB_2541995
FAM161A	Rabbit anti-FAM161A, Sigma-Aldrich (Atlas Antibodies), HPA032119-100UL	1:100	Primary antibody	AB_10602806
Anti-sheep Alexa 555	Donkey anti-Sheep Alexa 555, Thermo Fisher Scientific, A-21436	1:1000	Secondary antibody	AB_2535857
Anti-mouse Dylight 488	Donkey anti Mouse IgG (H + L) Cross-Adsorbed Secondary Antibody, Thermo Fisher Scientific, SA5-10,166	1:400	Secondary antibody	AB_2556746
Anti-rabbit Alexa 650	Donkey anti-Rabbit IgG (H + L) Cross Adsorbed Secondary Antibody, DyLight 650 conjugate, Thermo Fisher Scientific, SA5-10,041	1:400	Secondary antibody	AB_2556621
Hoechst	Hoechst 33,342, Trihydrochloride, Trihydrate, Thermo Fisher Scientific, H1399	1:2000	DNA Stain	AB_2651135

### Confocal microscopy

Slides were visualized using a Leica SP8 Confocal microscope in photon counting mode using a HC PL APO CS2 63 × 1.40 numerical aperture oil lens, 630× total magnification, format of 4096 × 4096 pixels (245 × 245 μm), 0.75 zoom factor, or with a format of 2048 × 2048 pixels (122.5 × 122.5 μm) 1.5 zoom factor, 2.0 frame accumulation, and three sequences:

Sequence one produces two images, one of DNA and the other phase-like. To capture DNA labeling via Hoechst 33,342, we used 0.1% 405 nm (UV) laser. Absorption spectrum was set to cover 410–478 nm via HyD1detector, which was set to standard, and was assigned a blue color. To create a phase-like picture, the PMT Trans was set to ON with a gain of 300. Fluro turret was set to Scan-PH (Phase).

Sequence two produces three images. To capture acetylated tubulin labeling via ALEXA 488, we activated a 488 nm laser set at 2.5% power. Absorption spectrum was set to cover 493–551 nm via HyD3 detector, which was set to counting, and was assigned a green color. To capture POC1B or FAM161A labeling via ALEXA 650, we activated a 633 nm laser set at 1% power. Absorption spectrum was set to cover 638–718 nm via HyD4 detector, which was set to counting, and was assigned a magenta color. This sequence produces a similar phase image to the first sequence. Sequence three produces two images. To capture tubulin labeling via ALEXA 555, we activated a 561 nm laser set at 0.75% power. Absorption spectrum was set to cover 566–623 nm via HyD3 detector, which was set to counting, and was assigned a red color. This sequence produces a similar phase image to the first sequence.

We collected multiple Z sections (10–20) of 0.3 μm thickness from the top of the highest sperm to the bottom of the lowest sperm. Images were taken with the photon counting mode, a gain of 10, and a laser intensity (~ 1%) that prevented signal saturation of the centriole biomarkers so we could record both increases and decreases of signal.

### Labeling quantification

We generated a max projection of all the layers of each image using the LASX program. The labeling intensity of each marker in the acquired images was quantified in the PC, DC, and Ax of each sperm using a 1.0 × 0.75 μm rectangular region of interest (ROI) using the Draw Rectangle tool in the Las X program (Fig. 1). The PC ROI is placed horizontally to the head where there is the most intensity. The DC ROI is placed perpendicular to the PC rectangle where there is the most intensity while the Ax ROI was positioned 2 μm away from the edge of the DC rectangle, downstream on the tail. Every sperm within an image was measured, regardless of sperm phenotype excepting sperm that had detached tails, those that were distorted to a point where normal ROI placement would have been impossible, sperm whose ROIs would overlap each other, or those that had artifacts in the ROIs. Quantification values were gathered by adding the maximum intensity values for each pixel within the ROI of channels 3, 4, and 6 which contain data from acetylated tubulin (green), either FAM161A or POC1B (magenta), and tubulin (red) respectively.

### FRAC test for subfertility

A FRAC test of individual bulls is ether positive or negative. A FRAC test is negative if all the 12 variables’ 95% confidence intervals are fully or partially within the reference range. A FRAC test is positive if any one of the 12 variables is an outlier (outside the reference range).

We perform a FRAC test of individual bulls in five steps.

1. *Intensity quantification*: We quantified the labeling intensity of each biomarker in the PC, DC, and Ax ROIs in each sperm (Fig. 1). These produced three intensity values per sperm. The quantification was blinded; the raters did not know which bulls were fertile or subfertile.
2. *Single sperm FRAC ratio*: For each biomarker (tubulin, acetylated tubulin, POC1B, and FAM161A) and location (PC, DC, or Ax), an excel sheet compared the max intensity pixel sum at a specific location to the total max intensity pixel sum of the three locations for each sperm.
3. *Sperm population mean FRAC ratio* (aka, mean FRAC ratio): We calculated the mean and 95% confidence interval of each of these FRAC ratios per sperm population (> 100 sperm), Because we analyzed four biomarkers and looked at three sperm locations, we obtained 12 variables for each bull population. Because we analyzed 2 populations of sperm for each bull, we obtained 24 variables for each bull.
4. *Reference range*: We determined the high-quality sperm population distribution range by calculating the *mean FRAC ratio* mean ± 2 standard deviations (SD) of the high-quality sperm of fertile bulls (having SCR above − 3). Sperm population mean ratio 95% confidence intervals that were partially to fully within the average mean FRAC ratio ± 2 SD were considered normal. 95% confidence interval mean FRAC ratios that were fully outside the boundaries of normal distributions were considered outliers and to have abnormal centriole labeling.
5. *FRAC test*: A bull with normal centriole labeling in all 12 parameters in high-quality sperm populations is considered to have a negative FRAC test. A bull with one or more outliers in any of the 12 parameters in its high-quality sperm population is considered to have a positive FRAC test. Positive FRAC tests are more severe with increasing number of outliers and increasing distance of the mean FRAC ratio from the reference range as determined by number of standard deviations.
6. Cohort FRAC tests: Cohorts were then compared to each other by comparing the ratio of outlier parameters out of total parameters in fertile and subfertile bulls (parameter-level FRAC test). Cohorts were also compared to each other by comparing the ratio of bulls with any outlier parameters out of the total number of bulls (individual-level FRAC test).

### Reliability

To gain insight into the reproducibility of the quantification step, we tested reliability in two ways.

First, we assessed rater performance by comparing the twelve mean FRAC ratios across four different pictures analyzed by the same three raters using intraclass correlation coefficient (ICC)^61^. Because we selected our raters from a large population of raters with similar characteristics (students with no prior experience), we used a “Two-Way Random-Effects” Model. This model allows us to generalize our reliability results to any raters who possess the same characteristics as the raters selected for the reliability study. As FRAC uses the mean ratio of multiple raters as the basis of measurement, we used the “Multiple Raters/Measurements” Type. Since we were concerned with different raters assigning the same score to the same subject, we used the “Absolute Agreement” Definition, culminating in ICC form (2,k) where 2 indicates a 2-way random effects model, and k indicates type: the mean of k raters. Then, we compared the raters to each other. Based on the 95% confidence interval of the ICC estimate, values less than 0.50 are indicative of poor reliability, values between 0.5 and 0.75 are indicative of moderate reliability, values between 0.75 and 0.90 are indicative of good reliability, and values greater than 0.90 are indicative of excellent reliability.

Second, sample to sample reliability of the FRAC analysis was also calculated by ICC (2,k)^61^. These calculations treated the different samples as raters and the different biomarker locations as subjects. As there were variable numbers of labelings for the different bulls, the value of k (equal to the number of labelings in this case) was not constant between the bulls.

### Statistical analysis

The normal distribution of the reference population’s *mean FRAC ratios* was determined after calculating the skewness and kurtosis by the functions SKEW and KURT in Excel. We also tested for normality (Shapiro–Wilk, D'Agostino-Pearson, Jarque–Bera, Cramer–von Mises, and Anderson–Darling) using https://www.gigacalculator.com/calculators/normality-test-calculator.php (used on date 5/27/2022) (Supplementary Table 1).

95% confidence intervals (AKA margin of error) of individual mean sample ratios were calculated using CONFIDENCE.T in Excel and were less than ± 0.04 from the mean in the high-quality sperm populations (Supplementary table 2 Summary sheet).

T-Tests were performed using the T.TEST function in Excel. Z-tests for two population proportions were calculated using the formula (1 − (NORM.DIST(ABS(((p̂_1_) − ((p̂_2_))/SQRT((p̂)*(1 − (p̂))*(1/(n_1_) + 1/(n_2_)))),0,1,TRUE)))*2 in excel.

We displayed numbers with 2 significant figures after the decimal point using normal rounding (i.e., 0.284 was rounded to 0.28 and 0.285 was rounded to 0.29). Pearson Correlation R and best fit were calculated by the function PEARSON(independent array, dependent array) and the automatic linear regression in Excel, respectively. The Pearson Correlation P-value was calculated using the function T.DIST.2T (absolute T value, degrees of freedom) in Excel. The Pearson Correlation R2 was calculated by the automatic linear regression in Excel. N was calculated using the functions COUNT and COUNTIF.

The T statistic was calculated using the equation (R*SQRT(N − 2))/(SQRT(1 − R^2)), and the degrees of freedom was calculated by N − 2, in Excel.

ICCs were calculated using the ICC function included in the Real Statistics Resource Pack for Excel.

Odds ratios were calculated using the formula (number of true positive * number of true negative)/(number of false negative * number of false positive). The Haldane-Anscombe correction was used for any groups that were zero^62^.

### Super resolution and expansion microscopy

#### Immunofluorescence labeling for stimulated emission depletion (STED) analysis

Bull sperm was purified as described above in the density gradient centrifugation, and the sperm suspension was added to a coverslip coated with 1 mg/mL poly-L-Lysine (Sigma-Aldrich; P5899), and incubated 3–5 min to allow sperm adhesion. Samples were fixed in 1.5% formaldehyde at RT for 4 min and post-fixed in 100% methanol at − 20 °C for 4 min, followed by rehydration in PBS. Samples were blocked in immunofluorescence (IF) buffer (1% BSA and 0.05% Tween-20 in PBS) for 15 min at RT. Samples were incubated with anti-acetylated tubulin antibody (Sigma-Aldrich; T7451) diluted 1:3000 in IF buffer for 24 h at 4 °C., followed by incubation in anti-mouse secondary antibody conjugated with STAR RED (Aberrior; STRED-1001) diluted to 1:200 for 2 h at 37 °C. DNA was labeled with 0.5 µg/ml DAPI (ThermoFisher Scientific; D1306) in PBS for 2 min at RT. Samples were embedded in mounting medium and imaged.

#### Sperm sample expansion and immunolabeling

Samples were expanded as previously described^63^. Coverslips containing immunolabeled sperm were postfixed in 4% formaldehyde in PBS at RT for 1 h and incubated at 40 °C for 16 h in a solution containing 30% acrylamide (Sigma-Aldrich; A4058) and 4% formaldehyde in PBS. Following three 10 min washes with PBS, coverslips were cooled in an ice-water bath. Precooled gelling mixture (20% acrylamide, 7% sodium acrylate (Pfaltz & Bauer; S03880), 0.04% bis-acrylamide (Sigma-Aldrich; A9926), 0.5% ammonium persulfate (Sigma-Aldrich; 248614), and 0.5% Tetramethylethylenediamine (Sigma-Aldrich; 411019)) was pipetted to the coverslips and incubated on ice for 20 min and 1 h at RT. After polymerization of the gel, smaller samples were excised using a 4-mm biopsy puncher (Integra Miltex; 33-34-P/25). And placed in an empty 50 ml conical tube and dry heated at > 90 °C for 10 min. Preheated SDS solution (200 mM SDS, 200 mM NaCl, 50 mM Tris, pH 9.0) was added to the punches, which were boiled for 1 h at > 90 °C. SDS solution with punches was cooled to RT. SDS was removed from punches by washing them extensively in PBS. To immunolabel acetylated tubulin, punches were blocked in IF buffer (1% BSA and 0.05% Tween-20 in PBS) for 1 h at RT and incubated with anti-acetylated tubulin antibody (Sigma-Aldrich; T7451) diluted to 1:3000 in IF buffer for 48 h at 4 °C. Punches were washed in PBS for 1 h and incubated with anti-mouse secondary antibody conjugated with Abberior STAR RED (Aberrior; STRED-1001) diluted to 1:50 in IF buffer for 24 h at 4 °C. Hoechst was added during immunolabeling to visualize DNA. Samples were expanded in deionized H_2_O and mounted in Rose chambers for imaging^63^.

#### Stimulated emission depletion (STED) microscopy

Imaging was performed with STEDYCON (Abberior Instruments) assembled on Eclipse Ti_2_ inverted microscope (Nikon Inc.), using 100×, NA 1.45 Plan Apo objective. Avalanche photo detectors (650–700 nm; 575–625 nm; 505–545 nm) were used to detect the signals. Browser-based control software (Abberior Instruments) was used to generate STED images. Images were acquired with a pinhole size of 32–65 µm and a pixel size of 10 nm. STAR RED was excited with 2–10% laser power and depleted with the STED laser at 97.88%. Signals were detected within a 7 ns gate.

#### Structured illumination microscopy (SIM)

SIM was performed on N-SIM, Nikon Inc., equipped with 405, 488, 561, and 640 nm excitation lasers, Apo TIRF 100 × NA 1.49 Plan Apo oil objective, and back-illuminated EMCCD camera (Andor, DU897). Images were reconstructed to generate a final image using Nikon NIS-Elements software.

## Results

### FRAC analysis of fertile and subfertile bulls used in AI industry

Sperm samples from thirty Holstein bulls and one Jersey bull were obtained from the Select Sires, Inc. population of bulls that have been used in artificial insemination (AI). These bulls’ SCR scores ranged from a minimum of − 18.2 to a maximum of + 2.8 (Supplementary Table 3). Fertile bulls were classified as those that have SCR deviations > − 3 and sub-fertile bulls are those that have SCR deviations < − 3^6,64,65^. All 31 bulls passed a rigorous semen and sperm analysis before they were used for artificial insemination in the field^6^. Therefore, it was surprising that six of them (five Holsteins and one Jersey) had an SCR that is significantly lower than the average and in the subfertile range. For our study, we divided the 31 bulls to two categories: (1) the fertile category included 25 bulls with SCR deviations above − 3 and (2) the subfertile category included six bulls with SCR at or below − 3. This is a ratio of about four fertile bulls for each subfertile bull, which is preferred in small control case studies^66^. To determine bull sperm centriole quality, we used a quantitative immunofluorescence-based assay named FRAC, which was developed to compare labeling intensity ratios in the proximal centriole, distal centriole and the axoneme and, thereby, relative protein levels in human sperm^21^ (Fig. 1)(Supplementary Table 2). Since the FRAC ratio is a relative number, increase in a FRAC ratio can be because of an intensity increase at that location or because of an intensity reduction in one of or both two other locations. Therefore, FRAC ratio in the Axoneme can go up if there is intensity reduction in the PC, DC, or both, or alternatively if a centriolar protein mis-localizes to the axoneme.

### FRAC experiments and raters are highly reproducible

The repeatability of FRAC tests is important for its reliability. Therefore, to gain insight into quantification reproducibility, we performed two types of evaluations.

First, we have compared three raters that analyzed the same four different pictures. Each picture included ~ 10 sperm for a total of 40 sperm per rater. Each rater generated nine averaged FRAC ratios per picture for a total of 36 values. We found the raters had an excellent intraclass correlation coefficient (ICC) of 0.9900 with 95% confidence interval between 0.994 and 0.998. Therefore, we concluded that FRAC have “excellent” inter-rater reliability (Supplementary Fig. 1).

Second, we assessed the overall reproducibility of independent labelings. In this study, we analyzed one straw from each bull by dividing its sperm to multiple coverslips. Then, we performed at least six independent labelings of high-quality sperm on six slides prepared from each straw. The first set of three slides were stained with tubulin, acetylated tubulin, and POC1B. Additional sets of three slides were stained with tubulin, acetylated tubulin, and FAM161A. Therefore, for this analysis, we compared the tubulin and acetylated tubulin of the three independent labelings that included POC1B to each other as well as to the three independent labelings that included FAM161A, instead of POC1B. This process was repeated for the high-quality sperm of all 31 bulls (Supplementary Fig. 2). We found excellent ICCs of more than 0.9400 for all bulls’ high-quality sperm. The 95% confidence intervals of 27 of the 31 ranged between 0.900 and 1, indicating an excellent inter-labeling reliability. The ranges of the remaining four bulls were between 0.840 and 1, indicating a good to excellent inter-labeling reliability.

#### Mean FRAC ratios of high fertility bulls’ high-quality sperm had a mostly Gaussian distribution

FRAC testing assumes statistical Gaussian (also known as normal) distribution, which is needed to establish that 95% samples are expected to fall within two standard deviations of the mean^67^. Therefore, we checked for normality using two tests: D’Agostino skewness test and Anscombe-Glynn kurtosis test^68^ and found that most of the mean FRAC ratios in the fertile bulls’ high-quality sperm population had near Gaussian/normal distribution (Supplementary Table 4).

In contrast, the distributions of FRAC values of for fertile bulls’ individual sperm mostly show narrow distributions around one peak that rarely exhibit Gaussian distribution (Supplementary Fig. 3). The skewness (the degree of asymmetry of a distribution around its mean) is smaller than + /− one for all the mean ratios of Acetylated tubulin and POC1B and most of mean ratios of tubulin (PC and Ax) and FAM161A (PC and DC). The skewness was larger in DC tubulin and axonemal FAM161A mean ratios (1.44 and 2.06). The skewness of axonemal FAM161A is likely because it is a centriole specific protein that is rarely found in the axoneme, and the distribution is capped at zero with values distributing only to its right (Supplementary Fig. 3L). Surprisingly, the skewness of DC tubulin mean ratios was also larger (~ 1.44) and indicated a skew toward the right (Supplementary Fig. 3E). The kurtosis (sharpness of the peak around the mean) is below + /− three for all the mean ratios of acetylated tubulin, tubulin and POC1B and most of mean the ratios of FAM161A (PC and DC). The kurtosis of axonemal FAM161A mean ratios was larger (5.14), probably because it is capped at zero (Supplementary Fig. 3L).

#### High fertility bulls’ high-quality sperm mean FRAC ratios have a narrow distribution with only 2% of the parameters and 16% of bulls being outliers

A good test should have a small reference range and a large effective dynamic range to identify deviation from normality. The effective dynamic range is defined as the range outside the smallest and largest values of the reference range. FRAC’s reference range is the average mean ratio plus and minus two standard deviations in the high-quality fertile sperm populations. We found that the reference range of fertile bulls’ high-quality sperm had different sizes for the various biomarkers in the three different locations, but it was always less 0.25 (Supplementary Fig. 4). Since FRAC ratios fall between zero and one, the effective dynamic range of the mean ratios for the data we have collected is, in all cases, at least three times the reference range. This is a remarkably narrow distribution which facilitates identifying centriolar anomalies with high sensitivity.

The average reference range for POC1B was relatively small (0.11), and high for tubulin (0.19), acetylated tubulin (0.20), and FAM161A (0.20) (Supplementary Fig. 4). This reduced variability appears to be because POC1B levels in the axoneme are consistently low. Interestingly, this is different than the reported average reference range in humans which reported higher average reference range for POC1B (0.15) and lower average reference range for tubulin (0.10) and acetylated tubulin (0.08) (Supplementary Fig. 4)**. This** may be due to lower general fertility in humans, a selection bias in either bovine or human samples, or that there is a species-to-species difference.

**Figure 2:**
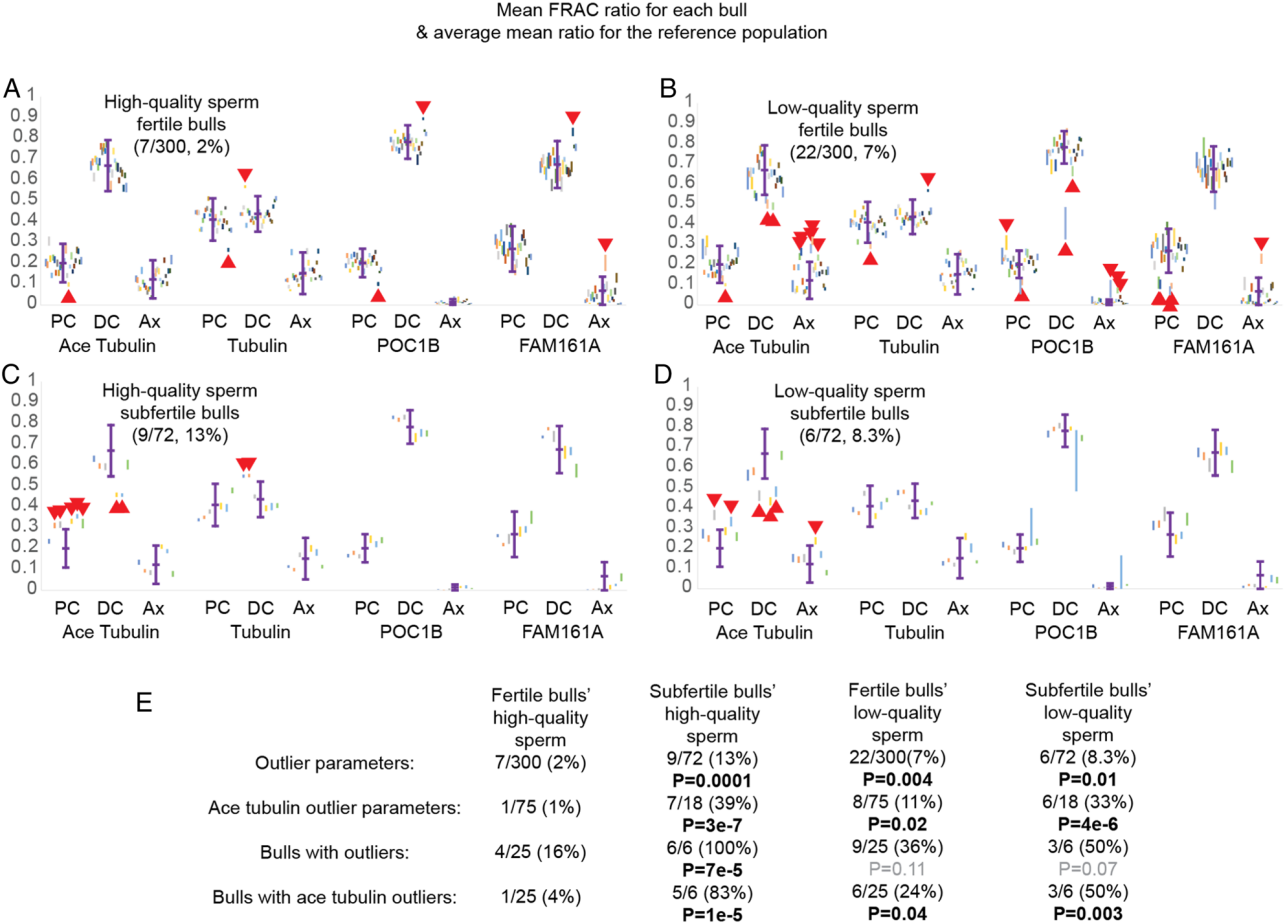
Sperm of subfertile bulls have abnormal centriole protein distribution. (**A**) The reference range (mean ± 2SD, in purple) for tubulin, acetylated (Ace) tubulin, POC1B, and FAM161A from the PC, DC, and Ax in fertile bulls’ high-quality sperm populations. This reference range (mean ± 2SD) is also depicted in (**B**–**D**) as a reference. Near each reference range, the individual FRAC ratio 95% confidence intervals from fertile bulls’ high-quality sperm are organized from left to right in order of increasing SCR. The y-axis represents the mean FRAC ratio. Fraction and percent of outlier values out of total values measured appear in the top left corner. (**B**) The 95% confidence intervals of fertile bulls’ low-quality sperm relative to the reference distribution. (**C**–**D**) The 95% confidence intervals of high-quality (**C**) and low-quality (**D**) sperm populations of subfertile bulls relative to the reference range. In (**A**–**D**), outlier values are indicated by red triangles. Data represent more than 25,000 stained sperm. (**E**) Different two proportion z-tests based on outlier data in (**A**–**D**). P-values are the result of comparing the column’s proportion to the proportion in the “Fertile bulls’ high-quality sperm” group. “Outlier parameters” counts the total number of outliers within a group out of the total number of parameters that were measured. “Ace tubulin outlier parameters” counts the number of acetylated tubulin outliers out of total acetylated tubulin parameters. “Bulls with outliers” counts the number of bulls within a group that have at least 1 outlier out of total number of bulls. “Bulls with ace tubulin outliers” counts the number of bulls within a group that have at least 1 acetylated tubulin outlier out of the total number of bulls. All raw data can be found in Supplementary Table 2.

Normally, a new test is compared to a gold standard test, a test that has been thoroughly tested and has a reputation in the field as a reliable method^69^. Because there is no “gold standard” to assess centriole quality, we use a reference range obtained from fertile bulls. An important feature of a good centriole-based test is that its reference range includes most of the high-quality centrioles for a given parameter. Indeed, we found that 21 of the 25 fertile bulls had all their 95% confidence intervals fall within the reference range. Only four of the 25 fertile bulls (16%) had mean ratio 95% confidence intervals outside of the high-quality sperm population’s reference range, making them outliers (Fig. 2A and E). These include bulls 10, 20, and 22 (SCR = − 1.5, 1, and 1.3, respectively) each had one outlier, while bull 29 (SCR = 2) had four outliers. Altogether, only 7 of the 300 analyzed parameters from high-quality sperm of fertile sires (2%) had mean ratio 95% confidence intervals outside of the high-quality sperm population 95% confidential interval (Fig. 2A).

#### Low-quality sperm of fertile bulls have abnormal centriole protein distribution

As sperm cells differentiate, they lose their cytoplasm and become denser; this distinction is used to separate the higher-quality sperm found in the pellet and the lower-quality sperm, which is found in the interface after differential centrifugation^70,71^. It is also during this differentiation that the sperm centrioles are remodeled^20^. Here, we analyzed the low-quality sperm population of the 25 fertile bulls (Fig. 2B). Nine of the 25 (36%) analyzed fertile bulls had at least one outlier parameter. However, this rate difference (9/25 versus 4/25) is not statistically significant (*P* = 0.11). When examining individual parameters, the low-quality sperm from fertile bulls had 22 parameters outside of the high-quality sperm population reference range, this rate difference (7/300 versus 22/300) is statistically significant (*P* = 0.004) (Fig. 2E). The latter result suggests the fertile bulls' low-quality sperm population has lower quality centrioles relative to the fertile bulls’ high-quality sperm. Similar lower centriole quality results were reported with sperm of infertile men^21^ but not in fertile men or men with unexplained infertility^33^.

### High-quality sperm of subfertile bulls return positive FRAC tests

We have analyzed five subfertile Holstein bulls and one subfertile Jersey bull (Fig. 2C). All the high-quality sperm populations of these subfertile bulls (6/6, 100%) had at least one mean ratio outside of the high-quality sperm distribution range of the fertile bulls’ high-quality sperm. This rate is much higher than high-quality sperm of fertile bulls (4/25, 16%), and is significantly different (*P* = 0.00008) (Fig. 2E). These six bulls’ high-quality sperm populations also had nine outlier parameters out of 72 parameters analyzed (13%). This is more than five times the rate of outliers observed in fertile bulls’ high-quality sperm (7/300, 2%), and is significantly different (*P* = 0.0001). This result suggests that subfertile bulls’ high-quality sperm population has inferior centrioles compared to fertile bulls’ high-quality sperm.

If we set a **cutoff** (aka threshold) of one FRAC outlier 95% confidence interval to indicate subfertility, the test is 100% sensitive, and 84% specific, which is a good diagnostic performance. A more stringent cutoff to indicate subfertility may be having two FRAC outlier 95% confidence intervals. Two outlier parameters were present in half of the subfertile bulls’ high-quality sperm populations (3/6, 50%). This rate is over ten times higher than high-quality sperm of fertile bulls (1/25, 4%) and is significantly different (*P* = 0.0025). Such a cutoff suggests a sensitivity of 50% and specificity of 96% for subfertility; these values must be considered carefully because of the small sample size.

None (0/6, 0%) of the subfertile bulls’ high-quality sperm populations had more than two outliers. However, one fertile bull did, (1/25, 4%) resulting in a larger proportion than in the subfertile population. This difference is not significant (*P* = 0.62). We suspect that this is due to having a smaller group of subfertile individuals, and that, if the sample size were larger, we would see individuals with more than two outliers in their high-quality sperm population.

To gain more insight into FRAC ratios of subfertile bulls, we examine the distributions of their single-sperm FRAC ratios (ratio distributions) (see graphs in Supplementary Table 5). As expected, most of the single bulls’ ratios are distributed around one peak (Supplementary Table 5). All subfertile bulls had an apparent shift in the distribution relative to the reference population (marked in yellow highlight), Bulls 1 and 2 in the tubulin PC parameter, Bulls 2–6 in acetylated tubulin in the PC, and Bulls 4 and 5 in acetylated tubulin in the DC.

However, Bull 6’s distribution had a long single tail toward the right with some additional smaller peaks of acetylated tubulin in the PC. Bull 6 also had sperm with abnormally high FRAC values in FAM161A in the PC and abnormally low FRAC values in FAM161A in the DC. This extended distribution suggests that Bull 6 has a sub-population of anomalous sperm.

Together, these observations suggest that the high-quality sperm population of subfertile bulls also have lower quality sperm centrioles than the high-quality sperm population of fertile bulls regardless of the type of comparison (number of bulls with an outlier or number of outlier parameters), as expected from our hypothesis. Overall, the data suggests that abnormal centriole protein distribution is common in the unexplained subfertile bulls. Therefore, selecting a bull with a negative FRAC test, may improve predictions of successful breeding.

### Subfertile bulls’ low-quality sperm population has inferior centriole protein distribution compared to fertile bulls’ high-quality sperm population

We analyzed the six subfertile bulls' low-quality sperm populations (Fig. 2D). Individual-parameter mean FRAC ratios outside of the reference range are found in both the low-quality sperm populations of subfertile bulls (6/72, 8.3%) and the low-quality sperm of fertile bulls (22/300, 7.3%), (*P* = 0.77) (Fig. 2E). Half (3/6) of the subfertile bulls’ low-quality sperm populations had multiple out-of-range parameters (from 2 of the 12 analyzed parameters), while a small percent of fertile bulls’ high-quality sperm population had multiple out-of-range parameters (1/26, 4%). This result suggests that subfertile bulls’ low-quality sperm population has inferior centriole protein distribution compared to fertile bulls’ high-quality sperm population (*P* = 0.002). Overall, the above data suggests that abnormal centriole protein distributions are common in subfertile bulls. Therefore, selecting a bull with high-quality centrioles may improve prediction of successful breeding.

### Acetylated tubulin is the most common biomarker of lower quality centrioles

We used two categories of centriole markers. Tubulin, POC1B, and FAM161A label structural proteins (tubulin labeled the centriole microtubule skeleton; POC1B and FAM161A label the centriole luminal scaffold that in the DC forms the rods). Acetylated tubulin is a post translational modification of tubulin. Therefore, these markers allow us to examine which of these distinct functional aspects are more sensitive for identifying subfertility. To identify marker sensitivity, we counted the number of outlier parameters found in each of the four sperm populations (Table 2). We found that acetylated tubulin had the highest number of outliers in total as well as in high-quality sperm from subfertile bulls, suggesting it has the best potential to identify subfertility.

**Table 2:** Acetylated tubulin is the most common marker of lower quality centrioles.

Marker	Location	High-quality fertile	Low-quality fertile	High-quality subfertile	Low-quality subfertile	Location total
Ace Tubulin	PC	1	1	5	0	7
	DC	0	2	2	3	7
	Ax	0	5	0	1	6
	Total	1	8	*7*	4	**20**
Tubulin	PC	1	1	0	0	2
	DC	1	1	2	0	4
	Ax	0	0	0	0	0
	Total	2	2	2	0	6
POC1B	PC	1	2	0	0	3
	DC	1	2	0	0	3
	Ax	0	3	0	0	3
	Total	2	7	0	0	9
FAM161A	PC	0	4	0	0	4
	DC	1	0	0	0	1
	Ax	1	1	0	0	2
	Total	2	5	0	0	7

The number of outlier 95% confidence interval FRAC ratios of the 31 bulls used in this study. Acetylated tubulin has the highest number of outliers in total (Bold) and highest number of outliers subfertile bulls in high-quality sperm (Italic).

### Acetylated tubulin in the PC correlates with SCR and subfertility

**Figure 3:**
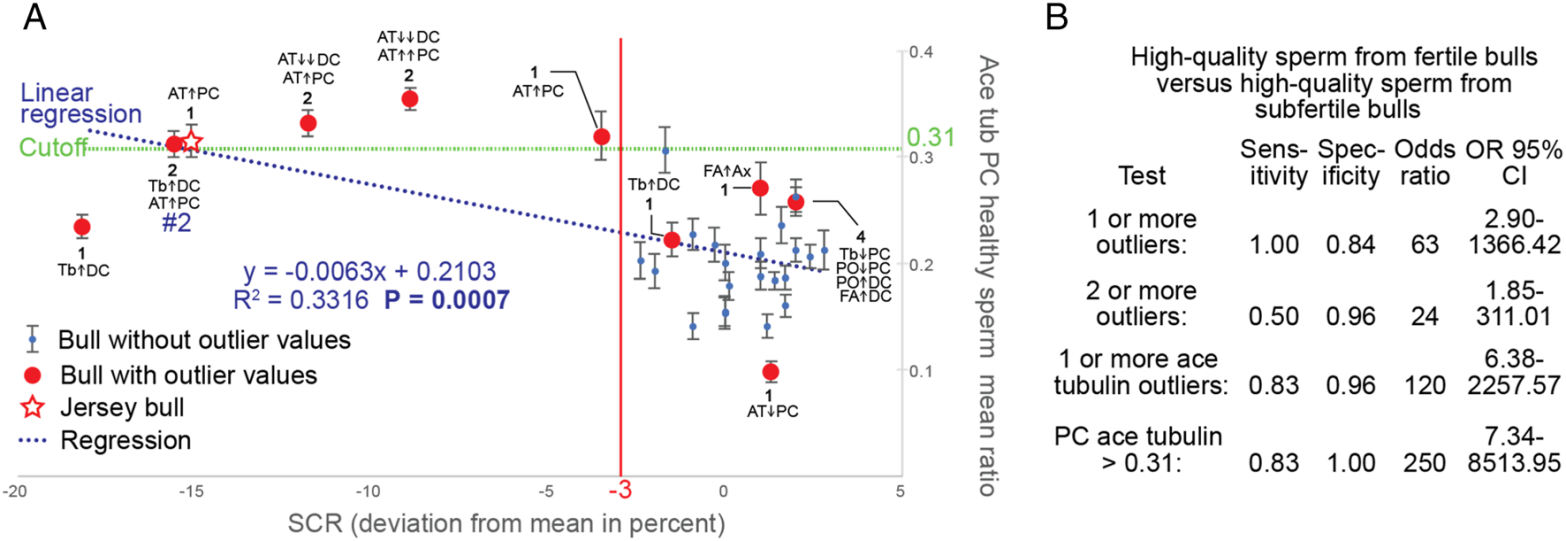
PC’s Acetylated tubulin correlates with SCR and subfertility. (**A**) A graph showing the correlation between a bull’s SCR deviation from the mean and the FRAC acetylated tubulin mean ratio in the PC of its high-quality sperm. Each bull is represented by a dot (blue for negative FRAC tests, and red for positive FRAC tests) as well as a 95% confidence interval. Bulls with outlier values are also marked with the name of the outlier biomarker (Tb, tubulin; AT, ace tubulin; PO, POC1B; FA, FAM161A), its location (PC, DC, or Ax), and arrows indicating if the outlier is higher (↑) or lower (↓) than the reference range. The number of arrows indicates the number of SD away from the reference range’s upper or lower bound (rounding up). Bull 2 (#2) which had an abnormal acetylated tubulin distribution is marked (see Fig. 4A). (**B**) Shown are the four tests, their sensitivity and their odds ratio used to determine fertility status in a bull. In the left column are the criteria each test uses to determine fertility status. In the far-right column are the 95% confidence intervals for the odds ratios (OR) shown in the third column. The test with the highest strength of association to accepted fertility status was based on the 0.31 cutoff generated by linear regression.

Because we found that acetylated tubulin most effectively identifies lower-quality centrioles (Table 2), we wondered if the mean FRAC ratios of acetylated tubulin have a quantitative correlation with SCR. We found a highly statistically significant (R = − 0.58; *P* = 0.0007) linear regression correlation between SCR and acetylated tubulin levels in the PCs of high-quality sperm (Fig. 3). This SCR correlation is much higher than any other reported variables from semen analysis, whose R values range between 0.10 and 0.16^6^. In PC Acetylated tubulin, the SCR score determined about one third of the variance observed (R^2^ = 0.3316). Based on this correlation, a cutoff of 0.31 in PC acetylated tubulin mean ratio identified five out of the six subfertile bulls and 0 of the 25 fertile bulls (Fig. 3B). This cutoff value has a sensitivity (the true positive rate) of 83% (5/6), and specificity of 100% (25/25) in the study population; however, these values must be considered carefully because of the small sample size. A weaker correlation was found in DC Acetylated tubulin (*P* = 0.02, R^2^ = 0.1719) and no correlation was found in Ax Acetylated tubulin (*P* = 0.90).

Interestingly, the odds ratio (the strength of association between test-proposed fertility status and accepted fertility status) is much higher in the test based on the 0.31 cutoff generated from the linear regression analysis than tests based on bulls’ positive FRAC test results (Fig. 3B).

Together, these data suggest that FRAC linear regression analysis can help identify bulls with lower sperm quality that are not recognized by current rigorous semen analyses.

### Anti-acetylated tubulin antibody marking the DC-Ax junction.

**Figure 4:**
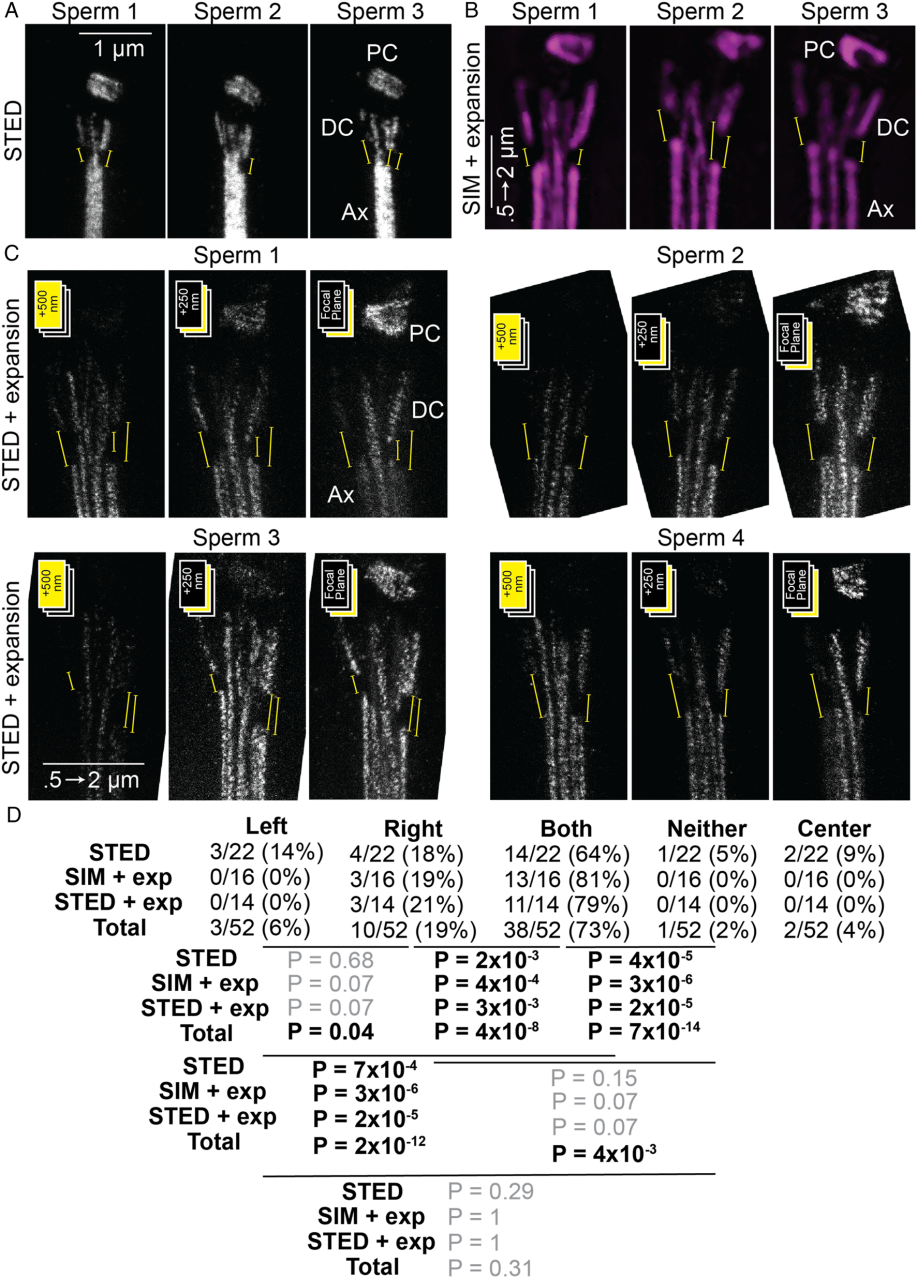
Anti-acetylated tubulin antibody discontinuously labels the outer DC microtubules. (**A**–**C**) Anti-acetylated tubulin signal is present on the microtubules of the centrosome complex (PC, DC, and Ax). Yellow lines mark the regions of outer microtubules in the DC-axoneme junction, where the acetylated tubulin signal is missing. In expanded sperm, the smaller number on the scale bar represents the size that the bar would have been before expansion. The larger number represents the real size after expansion. (**A**) Three cells imaged by STED. The scale bar of 1 μm applies to all panels. (**B**) Three 100 nm-thick Z-sections of expanded centrosome complex imaged by SIM. The scale bar of 2 μm corresponds to 0.5 μm pre-expansion. This scale bar applies to all panels. (**C**) Three 250 nm thick Z sections of four expanded centrosome complexes imaged by STED. The scale bar of 2 μm corresponds to 0.5 μm pre-expansion. This scale bar applies to all panels (**D**) The proportion of sperm that have gaps only on the left side (left), only on the right side (right), on both sides (both), on neither side (neither), and in the center (center) in the STED images, the SIM with expansion images, the STED with expansion images, and the combination of all images. P-values correspond to results of two-proportion Z-testing between sperm. Note that sperm have left–right asymmetry and the head is flat. All sperm were oriented with the proximal centriole on the right side for consistency and keeping the convention from our past studies.

Because acetylated tubulin immunolabeling was found to be a strong biomarker for subfertility, we examined its labeling pattern using stimulated emission depletion (STED) super-resolution microscopy (lateral resolution of ~ 30 nm) (Fig. 4A) and four times expansion microscopy in combination with structured illumination microscopy (SIM) (lateral resolution of ~ 40 nm) (Fig. 4B) or STED (achievable lateral resolution of ~ 10 nm) (Fig. 4C). All three approaches yielded similar results; acetylated tubulin signals were present in barrel-shaped PC, a fan-like DC, and the Ax. We presume that acetylated tubulin signals correspond to the microtubules.

Cryo-electron microscopy of the DC found that all the bovine sperm axonemes microtubules are continues with the DC microtubules^19^. Consistent with that, the acetylated tubulin immunolabeling central lines in the DC appeared continuous with Ax labeling in 96% of the analyzed sperm (Fig. 4D). These lines are probably the microtubule central pair of the Ax that extend into the DC. Unexpectedly, however, in most cases, at the junction of the DC and Ax, the acetylated tubulin signal appeared discontinuous in both the left and right sides of the DC (73% of the analyzed sperm) (Fig. 4D). This labeling gap suggests that there is a region of reduced acetylation of the outer microtubules at the DC-Ax junction.

## Discussion

Here, we found that the FRAC method can identify differences in centriole biomarkers between fertile and subfertile bulls, suggesting it is a high-throughput and sensitive way to identify centriole-based subfertility and infertility.

The standard paradigm used to evaluate the performance of a new test is comparing the results of the new test with the results of the existing gold standard test. However, there are cases, like sperm centriole evaluation, with no gold-standard test. In this case, measures other than accuracy are calculated, including event rates, relative risks, and other correlation statistics (Rutjes et al., 2007). The test also needs validation by clinicians and researchers to determine whether the new test results are meaningful in practice. A key point in the validation process is defining a threshold (cutoff) that would allow clinical use of the test with confidence. We developed FRAC testing and demonstrated that FRAC can detect differences between men with different infertility types^21,33^. It should be noted that some of the distributions of the mean ratios in the fertile bulls have a slightly skewed distribution, implying that they are not gaussian. However, increased skewness increases standard deviation, thereby increasing the reference range sizes generated. This actually decreases the rate of false-positives generated by FRAC testing. The use of other fertile and subfertiile bulls to test the efficacy of the reference ranges reported here is also underway.

Here, we performed a small retrospective cohort-control study and found that FRAC can suggest the presence of centriole-based subfertility in bulls that previously would have been used for artificial insemination, only to find that they had lower rates of confirmed pregnancy. Future studies should include a more extensive retrospective cohort-control study that will estimate relative risk and a threshold, which would then be used for further validation in a prospective study. As artificial insemination centers might be collecting from thousands of bulls every year, promoting extensive use of FRAC requires developing automated labeling, imaging, and image quantification, which is currently underway. It would also be important to determine the stability of the centriolar phenotype, if it changes with bull age or external factors such as heat stress.

An unexpected outcome of this study is that six out of the six subfertile bulls had positive FRAC tests, and five out of six subfertile bulls had high PC acylated tubulin—this is a very high proportion of anomaly for a single sperm structure, the centriole, out of many possible structures in sperm cells. There are two likely, non-mutually exclusive, explanations for this observation: (1) The standard semen analysis prescreen of bulls is effective at identifying most cases of subfertility before the semen is put on the market, but this analysis is unable to detect centriole abnormalities, thus enriching for centriole-based subfertility. Semen analysis in tandem with FRAC may reduce bulls coming onto the AI market only to be found to have unexplained subfertility. (2) FRAC testing is sensitive to sperm abnormalities beyond the centrioles and can report on causes that are not primarily in the centriole but do affect centriolar labeling. Centriole remodeling occurs during spermatogenesis; it is possible that if there are problems in spermatogenesis or sperm homeostasis, the centrioles might be reporting these problems.

One explanation for the presence of FRAC outliers in the fertile bull population is that their high SCR is due to having other fertility properties that are extraordinary and mask centriole impact. On average, a bull has about a 32% rate of impregnating a cow using AI (34.6% in^72^. This low overall rate may be influenced by other factors such as DNA fragmentation^73^, oxidative stress^74^, and capacitation status^75^). If a bull is much better than average for any of these factors, the bull's centriole defect may be masked. Therefore, it would be essential to perform a multifactor analysis of the sperm in the future to test this hypothetical explanation. Finally, another explanation for these results is that FRAC assessment may vary within a bull across collections, and in this study, we have examined an individual ejaculate.

We find acetylated tubulin is discontinuous in the junction between the axoneme and the tip of the DC in bovine; however, how this pattern of localization is generated and for what role is currently unknown. Interestingly, in human sperm, the junction between the axoneme and DC tip is labeled by CEP290^20^. In somatic cells, Cep290 is found in the transition zone located between centriole and cilia^76,77^, while in sperm cells, the transition zone migrates to the tip of the midpiece and for the annulus^78^. These observations suggest that the junction between the axoneme and DC represents a distinct functional domain which requires further studies.

It was recently shown that protein acetylation protects sperm from spontaneous acrosome reactions^79^. Tubulin acetylation is a marker of stable microtubules, and its high level in the PC and DC is consistent with their microtubules’ stability. One of the dramatic findings in this study is that acetylated tubulin has the highest difference between fertile and subfertile bulls and has abnormally high levels in the PC of subfertile bulls. A similar trend was found in humans with unexplained infertility^33^. This may be because acetylated tubulin is a post-translational modification that can vary, while the other markers used, tubulin and POC1B, are structural proteins and likely more consistent. Acetylated tubulin may be more variable because post-translational modifications are less permanent and can change quickly in response to signaling or homeostatic changes in the sperm cell activating acetylases and deacetylases. In mammals, acetylated tubulin levels are mainly governed by opposing actions of α-tubulin acetyltransferase 1 (ATAT1) and histone deacetylase 6 (HDAC6)^80^, both of which are found in the sperm^81,82^. It is important to note that sperm are transcriptionally and translationally silent, and these modifications can allow sperm to respond to stimuli^83^ Reviewed in^84^.

Overall, FRAC has the potential to be a method of identifying subfertile bulls before use in artificial insemination, saving time and money for both artificial insemination companies and cattle breeders. Furthermore, these results also support our previous findings using FRAC to evaluate human sperm centriole quality. An additional method of male fertility evaluation yields more information when couples are deciding whether or what assisted reproductive technologies to utilize and may open avenues for treatment.

## Conclusion

FRAC could help identify subfertile bulls, improving artificial insemination success rates, and increasing cattle industry reproductive efficiency.

### Supplementary Information

Supplementary Information.

Supplementary Table 1.

Supplementary Table 2.

Supplementary Table 4.

Supplementary Table 5.

## Data Availability

The raw data underlying this article are available in the article and in its online supplementary material.
