# Supplementary material for: Abnormal centriolar biomarker ratios correlate with unexplained bull artificial insemination subfertility: a pilot study
Source: Sci Rep. 2023 Oct 26;13:18338. doi: 10.1038/s41598-023-45162-8 (PMC10603076; doi:10.1038/s41598-023-45162-8)
Supplement: Supplementary file 1 — Supplementary Information. [file 41598_2023_45162_MOESM1_ESM.pdf]

Supplementary Figure Legend

1. BOX 1. Definitions.

Centriolar biomarker – Either of the antibodies against Tubulin, Acetylated tubulin, POC1B, and FAM161A.

Fraction – The two groups of sperm generated by differential centrifugations: pellet (high quality sperm) and interface (low-quality sperm).

Mean ratio (aka the mean immunostaining intensity ratio) – the mean of the ratios for all sperm in a population from one bull at one location for one marker, usually calculated from more than 50 sperm. Each bull had up to 12 mean ratios (PC tubulin, DC tubulin, Ax tubulin, PC POC1B, DC POC1B, and Ax POC1B in the pellet and additional 12 in the interface).

Ratio – The ratios between the staining intensity of one marker in one location (e.g., PC) over the sum of the intensity of the same marker in all other locations in the same individual sperm (i.e., PC/PC + DC + Ax).

Ratio distribution – The distribution of single sperm ratios from a single sperm fraction from a single bull.

Reference population – The healthy sperm fraction (pellet) from the 25 fertile bulls (SCR >-3).

Reference range – The mean ± 2SD of the reference population for a marker at a given location (i.e., inside 95% confidence interval).

Sample (or semen sample) – the ejaculate or part of it obtained from a bull.

Sperm location – The three sites in the sperm used when quantifying marker intensity: PC, DC, and Ax.

Parameter – a category separating FRAC values by both centriolar biomarker type and sperm location

Sub-optimal centrioles – A bull who had at least one of its 12 mean ratios fall outside the reference range generated by fertile bulls' high-quality sperm.

2. Supplementary Table 1 – Normality of the mean ratio distribution

This file contains skewness and kurtosis values for the distributions generated by the mean ratios within a given parameter. The analysis for different groupings (i.e., fertile bulls, subfertile bulls, and all bulls) of bulls have been separated into different sections highlighted in red, green, white, and blue. Analysis pertaining to subfertile bulls' high-quality sperm has a red highlight, analysis pertaining to fertile bulls' high-quality sperm has a green highlight, and analysis pertaining to all 31 bulls' high-quality sperm has a white highlight. The raw data that the other sections use to make calculations is highlighted in blue. Skewness and kurtosis values were highlighted in red if they had a magnitude higher than 1 or 3 respectively. Also included are the average mean ratio, standard deviation, and 95% confidence intervals for the "subfertile bulls", "fertile bulls", and "all bulls" sections. Results for Shapiro Wilk, D'Agostino-Pearson, Jarque-Bera, Cramer-von Mises, and Anderson-Darling tests for normality are also included in the "fertile bulls", and "all bulls" sections.

See supplementary file: 2023 01 19 Supplementary Table 1 Turner Achinger et al.xlsx

3. Supplementary Table 2 – The FRAC data set

This file contains a summary of the FRAC data, and all the raw data collected. A summary of the FRAC data is present in the Sheet titled “Summary” and it presents mean FRAC ratios, number of sperm collected, standard deviation, 95% confidence interval, and the range of the confidence interval for each parameter studied. The raw data collected for each bull’s high-quality sperm, is presented in separate sheets.

This table is a large data set. See supplementary file: 2023 02 28 Supplementary Table 2 Turner Achinger et al.xlsx

Category	Bull ID	Deviation
subfertile	Bull 1	-18.2
subfertile	Bull 2	-15.6
subfertile	Bull 3J	-15.1
subfertile	Bull 4	-11.8
subfertile	Bull 5	-8.9
subfertile	Bull 6	-3.5
normal	Bull 7	-2.4
normal	Bull 8	-2
normal	Bull 9	-1.7
normal	Bull 10	-1.5
normal	Bull 11	-0.9
normal	Bull 12	-0.9
normal	Bull 13	-0.3
normal	Bull 14	0
normal	Bull 15	0
normal	Bull 16	0
normal	Bull 17	0.1
normal	Bull 18	1
normal	Bull 19	1
normal	Bull 20	1
normal	Bull 21	1.2
normal	Bull 22	1.3
normal	Bull 23	1.4
normal	Bull 24	1.6
normal	Bull 25	1.7
normal	Bull 26	1.7
normal	Bull 27	2
normal	Bull 28	2
normal	Bull 29	2
normal	Bull 30	2.4
normal	Bull 31	2.8

4. Supplementary Table 3 – Sample bulls' SCR distribution

This table contains Sire Conception Rate (SCR) data from all 31 bulls used throughout this paper numbered 1-31 in order of increasing SCR. After bull 6 is a solid black line, which denotes the cutoff between subfertile and fertile bulls. Bull 3J has a J in its ID number to denote that it is a Jersey bull rather than a Holstein.

5. Supplementary Table 4 – Distribution of the fertile bull FRAC data set

This file contains the distributions of the FRAC values of all sperm from each fertile bull. Each sheet is separated by parameter and contains both the distribution generated by graphing all the FRAC values from each high-quality sperm from our fertile bulls in an aggregated distribution (shown in blue) and the ratio distributions of the high-quality sperm from each individual bull (shown in red). Each graph represents the ratio distribution of an individual bull's sperm compared to the aggregate ratio distribution and has 10 bins that span from 0 to 1 in 0.1-unit intervals.

This table is a large data set. See supplementary file: 2023 03 01 Supplementary Table 4 Turner Achinger et al.xlsx.

6. Supplementary Table 5 – Distribution of the subfertile bulls FRAC data set

This file contains the distributions of the FRAC values of all sperm from each subfertile bull. Each sheet is **separated by parameter and contains both the distribution generated by graphing all the FRAC values from each high-quality sperm from our fertile bulls (shown in blue) and the ratio distributions of the high-quality sperm from each individual bull (shown in red). Also, each** sheet has graphs on the right side that represent each subfertile bull (in red) relative to the reference population (in blue). Note that in the sheet "ACE TUB PC", Bull 6 has a complex distribution with a most sperm in bins 0.05-0.25 and some relative increases in bin 0.45 and 0.75. Similar deviations in Bull 6 distributions graphs are observed in sheet FAM161A PC and FAM161A DC. Yellow highlight of the graph title indicates parameters that were found to be outlier values by FRAC.

This table is a large data set. See supplementary file: 2023 04 07 Supplementary Table 5 Turner Achinger et al.xlsx**.**

	Picture 1			Picture 2			Picture 3			Picture 4		
	Rater 1	Rater 2	Rater 3	Rater 1	Rater 2	Rater 3	Rater 1	Rater 2	Rater 3	Rater 1	Rater 2	Rater 3
Tubulin PC	0.46	0.41	0.48	0.39	0.4	0.41	0.43	0.45	0.43	0.41	0.41	0.42
Tubulin DC	0.39	0.45	0.36	0.43	0.42	0.42	0.40	0.40	0.38	0.43	0.42	0.42
Tubulin Ax	0.16	0.15	0.15	0.18	0.18	0.18	0.17	0.16	0.18	0.17	0.17	0.17
Ace Tub PC	0.18	0.22	0.2	0.26	0.27	0.27	0.20	0.21	0.21	0.14	0.13	0.13
Ace Tub DC	0.65	0.64	0.59	0.57	0.56	0.56	0.60	0.61	0.54	0.69	0.70	0.69
Ace Tub Ax	0.17	0.14	0.21	0.17	0.19	0.17	0.21	0.19	0.25	0.17	0.17	0.18
FAM161A PC	0.36	0.29	0.39	0.32	0.29	0.35	0.34	0.34	0.35	0.34	0.33	0.34
FAM161A DC	0.63	0.7	0.6	0.67	0.7	0.64	0.65	0.63	0.63	0.63	0.64	0.63
FAM161A Ax	0.01	0.01	0.01	0.01	0.01	0.01	0.01	0.02	0.02	0.03	0.03	0.03

ICC = 0.99 (Excellent)

7. Supplementary Figure 1 – FRAC raters' reliability is high

Three raters analyzed four pictures with ~40 sperm in total of a bull with SCR deviation of 1.7. Intraclass correlation coefficient with two-way random-effects model, single measurement, and absolute agreement. The ICC score is 0.99 (Excellent).

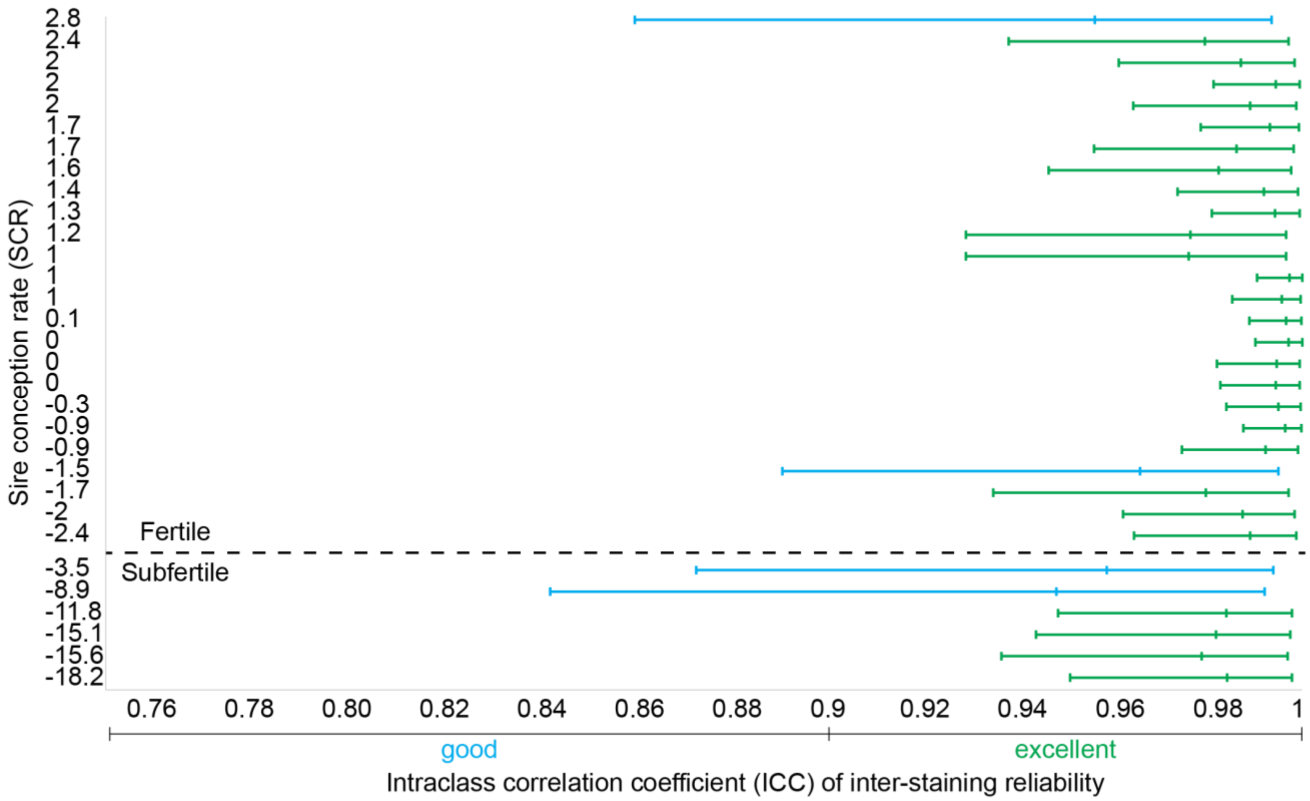

Supplementary Figure 2 - Reproducibility of independent FRAC tests is high.

We compared tubulin and acetylated tubulin mean ratios from 31 bulls with six or more independent stainings using two-way random-effects model, single measurement, and absolute agreement. 27/31 95% confidence intervals fell entirely within 0.900 and 1 (green), indicating excellent reliability. 4/31 confidence intervals fell between 0.840 and1 (blue), indicating good to excellent reliability.

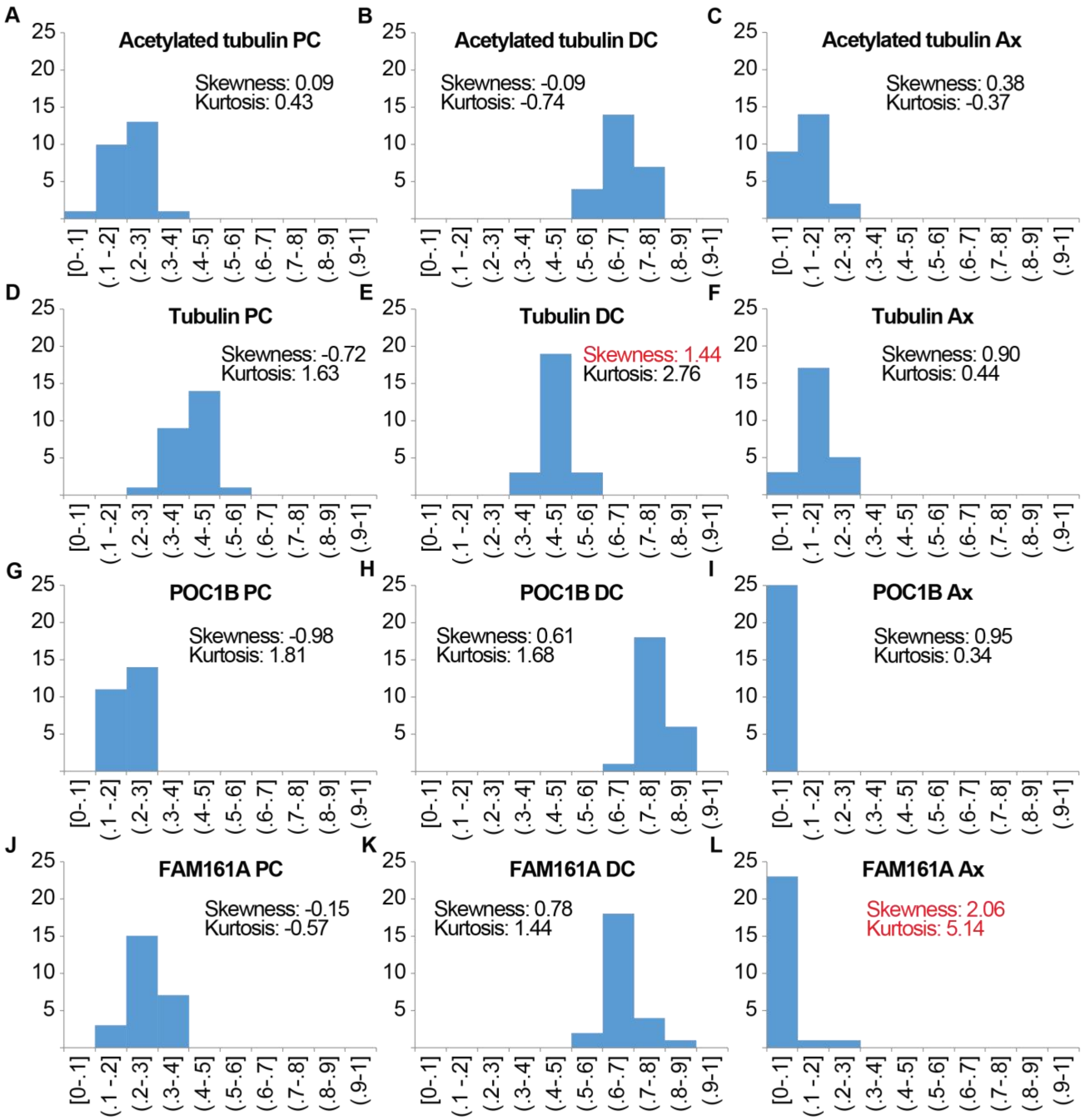

8. **Supplementary Figure 3 - High-quality sperm from high fertility bulls have mean FRAC ratios with a mostly Gaussian distribution.**

(**A-L**) Histograms of the 12 parameters within the reference population. The X-axis represents the boundaries of each of the ten bins with square brackets signifying that the bound is included within the bin, and curved brackets signifying that the bound is not included in the bin. The Y-axis represents the number of bulls that fall within each bin. When testing for skewness and kurtosis within the reference population, we found that two of the twelve parameters' skewness and/or kurtosis values fell outside of what would be considered normal (highlighted in red).

		Ace Tub	Tubulin	POC1B	FAM161A
Bovine	PC	0.18	0.20	0.14	0.22
	DC	0.24	0.17	0.16	0.23
	Ax	0.18	0.20	0.03	0.14
	Avg	**0.20**	**0.19**	**0.11**	**0.20**
Human	PC	0.03	0.10	0.12	ND
	DC	0.11	0.07	0.16	ND
	Ax	0.11	0.14	0.17	ND
	Avg	**0.08**	**0.10**	**0.15**	ND

9. Supplementary Figure 4 – High-Quality Fertile bull sperm distribution is narrow

The distribution of fertile bulls' high-quality sperm mean FRAC ratios (the upper bound minus the lower bound, ±2SD) in bulls (this study) and confirmed human fathers (from Jaiswal et al., 2022).
